# Effect of Protein Supplementation on the Gut Microbiome of Omnivorous and Herbivorous Goliath Beetles

**DOI:** 10.3390/insects17080871

**Published:** 2026-08-21

**Authors:** Pei-Rui Wu, Matan Shelomi

**Affiliations:** Department of Entomology, National Taiwan University, Taipei 106319, Taiwan

**Keywords:** digestion, insect-microbe interactions, microbial ecology, microbiology, Scarabaeidae, symbiosis

## Abstract

Scarab beetles depend on gut microbes for digestive enzymes. Some species’ microbiomes show taxonomic conservation regardless of diet, while others have conserved functional profiles. We compared the hindgut microbiomes of two Cetoniinae beetle larvae: the obligately saproxylophagous *Mecynorrhinella poggei* and the omnivorous/predatory *Goliathus goliatus*, which requires protein supplementation when reared artificially. Two diets with and without supplementation were used. If diet drives the microbiome, then gut microbes in protein-supplemented hosts should produce fewer lignocellulolytic enzymes and more proteinases regardless of species. If microbiome composition is conserved within a species, then *Mecynorrhinella* is expected to have more lignocellulolytic microbes while *Goliathus* should have more proteinolytic microbes regardless of diet. In this study, low-protein diets reduced *G. goliatus* growth, but gut microbiome composition and predicted function remained largely stable, dominated by Bacteroidales including *Dysgonomonas*, *Proteiniphilum*, and *Alistipes*. *Mecynorrhinella*’s gut microbiome showed some reduced *Proteiniphilum* and increased *Dysgonomonas* relative abundance, but otherwise the microbiome composition was statistically stable with no effect of diet on growth or predicted microbiome function. These results highlight that microbiomes of closely related insects can markedly differ even if diet does not and that microbiome functional conservation tied to host physiology may occur even if greater plasticity could theoretically reduce malnutrition.

## Simple Summary

Some insects like scarab beetles must get essential gut microbes from the environment to survive. Whether the gut microbiome is affected more by diet or by species-specific gut parameters is unknown for many species. In this study, the gut microbiomes of two species of scarab beetle larvae were compared: one that eats wood and one that must also eat meat, or at least protein-supplemented wood. Regardless of which diets either species fed on, the most abundant microbes in the larvae changed relatively little, and neither did their predicted functions. Clear differences existed between each species–diet pair and between species, but not between diets. Thus, both species of scarab beetle larvae in this study have mostly conserved gut microbiomes and predicted functional profiles, even if this causes one species to suffer protein deficiency due to a non-adaptive gut microbiome.

## 1. Introduction

Many insects rely on their gut microbes in whole or in part to digest their food. Herbivores may benefit from microbe-derived cellulase and other plant cell wall-degrading enzymes (PCWDEs) to convert wood polysaccharides into digestible sugars [1], which also releases other nutrients like nitrogen and phosphorus locked behind the cell walls [2]. Insects that feed exclusively on dry wood will be more dependent on a source of PCWDEs than those that feed on plant matter richer in accessible and digestible carbohydrates like fruits or detritus [3,4,5,6]. In fact, the current hypothesis is that saproxylophagy [feeding on dead wood] evolves from detritivory in part when detritivores gain fiber-degrading symbionts [7].

The comparative role of endogenous and microbial enzymes involved in nitrogen metabolism in herbivores is far less studied. Saproxylophages may require nitrogen-fixing microbes to mitigate the natural paucity of nitrogen in dead wood [8,9,10]. In theory, insects that supplement a mostly plant-based diet with animal protein, such as by opportunistically scavenging or predating on other invertebrates, should be less reliant on microbial nitrogen fixers and maybe less reliant on endogenous or microbial PCWDEs to maximize released nitrogen, while predators may not require PCWDEs or microbial nitrogen fixation at all [3,11,12]. On the other hand, many insects’ gut microbes significantly contribute to protein digestion beyond what the host’s endogenous enzymes are capable of. While less studied than PCWDEs, bacterial proteinases have been identified as significant contributors to host digestion in wood-feeding beetle larvae [13], herbivorous caterpillars [14,15], omnivorous black soldier fly larvae [16], and an omnivorous mole cricket [17]. The relationships insects have with their gut microbes when it comes to protein digestion are presently not well understood; whether wood feeders or omnivores will have more proteinolytic microbes is not hypothesizable.

The open question of “what determines the composition of the gut microbiome?” is particularly salient for insects that depend on environmental microbes to survive but lack vertical transmission of microbes from parent to offspring. Scarab beetles (Coleoptera: Scarabaeidae) are one such insect that appears not to have a conserved gut microbiome, but instead their gut is selectively colonized by a subset of microbes from those present in their diet [2,18]. Those that are PCWDE-producing at least are nonetheless essential, as scarabs do not produce active PCWDEs themselves [11,19]: their presence in the scarabs represents a host–obligate mutualism with a polyculture of flexible membership [20]. For example, work with the obligate wood-feeding larvae of the coconut rhinoceros beetle, *Oryctes rhinoceros* (Dynastinae), found that their hindgut microbiome enriches certain PCWDE-producing microbes from the decaying wood they consume, even if these are relatively uncommon in the wood itself [18,21,22,23]. Microbiome analysis of scarab beetles almost always finds a diversity of PCWDE-producing microbes and frequently also microbes capable of nitrification, denitrification, or nitrogen fixation, as noted in *Cetonia aurataeformis* (Cetoniinae) [2], *Copris incertus* (Scarabaeinae: Coprini) [24], *Costelytra brunneum* and *Costelytra giveni* (Melolonthinae) [25], *Cotinis nitida* (Cetoniinae) [26], *Maladera insanabilis* (Sericinae) [27], *Melolontha hippocastani* (Melolonthinae) [28], *Pachysoma endroedyi* and *Pachysoma striatum* (Scarabaeinae) [29], *Popillia japonica* (Rutelinae) [30], and *Protaetia acuminata* (Cetoniinae) [31].

The same phenomenon is likely in other, obligately saproxylophagous scarabs, such as the larvae of *Mecynorrhinella poggei* (Cetoniinae: Goliathini). Also within the Goliathini is *Goliathus goliatus,* a species highly prized by collectors and whose larvae are even eaten in some cultures [32]. However, its larvae are considered obligately omnivorous or even predatory, which is unusual for this subfamily [33]. Both *Goliathus* and *Mecynorrhinella* are extremely large beetles popular among breeders, and the many breeding manuals for these all agree that *Goliathus* requires a relatively protein-rich diet to survive, for example, by supplementing the typical rotten-wood substrate of Cetoniinae larvae with pet food [33]. Data on the gut microbiomes of these two closely related species is completely lacking, and multiple hypotheses are plausible for the composition and function of their microbiomes. Both are expected to have PCWDE-producing microbes, but the omnivorous *Goliathus* could potentially survive with fewer, as its diet is more nutritious. Alternatively, it may be less able to recruit PCWDE-producing microbes from its environment than *Mecynorrhinella*, and this in turn means it cannot survive on wood alone. Both explanations would suggest *Mecynorrhinella* has a more conserved complement of PCWDE-producing microbes; the hypotheses differ in whether diet drives the microbiome composition or the possible microbiome composition drives the diet. Regarding microbes involved in protein metabolism, one could hypothesize *Mecynorrhinella* needs more to account for the relative lack of protein in its diet, especially nitrogen fixers, or one could hypothesize *Goliathus* needs more to account for the relative abundance of protein in its diet, especially proteinase producers.

Also untested is what would happen to the microbiomes of these species if their artificial diets were switched: the protein-dependent *Goliathus* being reared on a wood-only diet and *Mecynorrhinella* reared on a protein-supplemented diet. While this is unlikely to happen in nature, such a diet switch allows us to test the hypothesis of whether these scarabs’ gut microbiomes always reflect their diet, with more proteinolytic or fewer nitrogen-fixing microbes in the guts of either beetle species if fed the protein-supplemented diet, or whether the microbiomes show functional and/or taxonomic conservation within each species, with PCWDE-producers and nitrogen-fixing microbes always more abundant in *Mecynorrhinella poggei* regardless of the insect’s diet [20]. Work outside of Scarabaeidae provides precedent for dietary changes affecting microbiome composition and function: the microbiome of the cockroach *Diploptera punctata* has more cellulolytic and nitrogen-fixing families when the insect is fed a cellulose-supplemented diet for four weeks and more proteolytic anaerobes when fed an animal protein-supplemented diet [34,35].

This paper compares the microbiomes of these two beetles fed either their appropriate diet or that of the other. If one hypothesizes that the insect’s dietary needs drove the evolution of its gut physiochemistry such that it selects the right microbes from the environment to survive, then one expects the gut of *Mecynorrhinella* will select for the necessary PCWDE-degrading and/or nitrogen-fixing microbes even in a diet higher in nutritionally superfluous proteins, while *Goliathus*’s microbiome will not be colonized by PCWDE-degrading and/or nitrogen-fixing microbes even if these would be beneficial [though not sufficient] for digestion. Alternatively, if one hypothesizes that the diet drives the microbiome, then one expects the gut of either species will have more proteinase-producing microbes when fed a higher-protein diet and/or more PCWDE-degrading and nitrogen-fixing microbes when fed a low-protein diet, even if these are not sufficient to allow the insects to thrive. It is further possible that one species’ microbiome is more diet-driven and another is more conserved; that these species are both in the same subfamily and tribe should not imply their microbial ecology is the same.

## 2. Materials and Methods

### 2.1. Insect Rearing and Growth Analysis

Adult *Mecynorrhinella poggei* and *Goliathus goliatus* were acquired from private breeders online in Taiwan and allowed to produce eggs. Larvae were reared in plastic containers filled with Insect Research A5 Fermented Wood Substrate (Insect Mall, Taipei, Taiwan), which is made of a mix of *Liquidambar formosana* (Formosan sweet gum) and other broadleaf hardwoods previously used to rear mushrooms. The source of protein was Ailin No. 4 Koi Fish Feed (Fwusow Industry Co., Ltd., Taichung, Taiwan), which is made primarily of fish meal and contains ≥ 34% protein, ≥4% fat, and ≤3% fiber.

For this study, from each species, four first-instar larvae were placed in individual containers containing one of two diets: 100% wood, or 2:1 wood and koi feed by volume. This sample size has precedence in other scarab beetle gut microbiome studies that also used sample sizes of four [36,37] or even three [24,38,39,40]. A negative control container of wood substrate without larvae or koi feed was also set up. The koi-supplemented substrate was prone to mold growth, which could be minimized or prevented entirely by leaving the koi pellets as solid balls rather than pulverizing and mixing them into the substrate. Once larvae reached the second instar, they were taken to the Department of Entomology at National Taiwan University. Measurements taken were larval mass using a laboratory balance (uncertainty ± 0.01 g), body width from the center of the body using digital calipers (±0.01 mm), and length from the back of the head capsule to the tip of the anal segment using a ruler (±0.1 cm). Means were statistically compared using a one-tailed, unpaired Student’s *t*-test.

### 2.2. Microbiome Sampling and Metabarcoding

To sample the gut microbiome, the three largest larvae of each set of four were surface sterilized by shaking in 70% ethanol for two minutes, then rinsed twice in deionized water. Then, under a laminar flow hood, the larvae were dissected using flame-sterilized tools. The distinctive and highly swollen hindgut paunch, which is physically separated from the midgut and from the anus by long and quite thin segments of digestive tract [22], can be easily cut apart from other gut sections. These were removed and placed into pre-weighed bead beating tubes of the Presto™ Soil DNA Extraction Kit (Geneaid Biotech Ltd., New Taipei City, Taiwan) and weighed. DNA was extracted following the kit’s procedures, eluting in water, and the DNA was purified using a Zymo Research^®^ OneStep™ PCR Inhibitor Removal Kit (Irvine, CA, USA). This sample size is suitable for an experiment looking for only broad changes in microbiome among large-bodied, slow-growing, and difficult-to-acquire organisms such as these beetles and falls within precedent for metabarcoding study sample sizes [40,41,42]. To determine the environmental microbiome, triplicate samples of 250 mg of the original wood substrate and koi feed pellets taken at the start of the experiment, as well as duplicate samples of 250 mg of the substrates in which the larvae had lived with and without koi feed supplementation and the substrate kept with no larvae, were used with the Presto™ Soil DNA Extraction Kit, pooled, and purified with OneStep™.

To identify the microbiome, metabarcoding was used. Samples from each larva and pooled samples of the other sources were sent to Tools Biotech for Illumina MiSeq sequencing of the V3/V4 region of the 16S rRNA gene (forward primer with the adapter region underlined: 5′-TCG TCG GCA GCG TCA GAT GTG TAT AAG AGA CAG CCT ACG GGN GGC WGC AG-3′; reverse primer with the adapter region underlined: 5′- GTC TCG TGG GCT CGG AGA TGT GTA TAA GAG ACA GGA CTA CHV GGG TAT CTA ATC C-3′) [43]. Data was analyzed using QIIME 2 v2025.10 [44,45]. Denoising was done with deblur [46], discarding features that only appeared in one sample. Taxonomic classification of operational taxonomic units (OTUs) was done using the Silva 138 99% OTUs classifier [47,48]. After rarefaction, the Bray–Curtis, Jaccard, and weighted UniFrac beta diversity metrics were calculated based on the feature table solely for the hindgut samples, and Principal Coordinate Analysis (PCoA) was performed on the results to visualize community structure in a reduced-dimensional space using R version 4.2.0 [49,50,51]. Values were compared with PERMANOVA and PERMDISP [52]. Differential abundance testing was done comparing the microbiomes of the four groups of beetles (two species x two diets) and between the two species regardless of diet using Analysis of Compositions of Microbiomes with Bias Correction (ANCOM-BC) with ancombc v2.x [45,53,54,55].

Predictive functional analysis was done using PICRUSt2 v2024.5 [56]. Using QIIME 2 version 2024.5, the full pipeline of PICRUSt2 was run on the full dataset of representative sequences [45,57]. The metagenome data for Enzyme Commission (EC) numbers, Kyoto Encyclopedia of Genes and Genomes (KEGG) orthologs, and MetaCyc pathways were exported as tsv files [55]. Beta- and alpha-diversity matrices were compared with PERMANOVA, and log fold changes were compared using ANCOM-BC as above [45,53,54]. Heatmaps were made using the pheatmap package of R v4.2.0 [58].

## 3. Results

### 3.1. Insect Development on Different Diets

**Figure 1:**
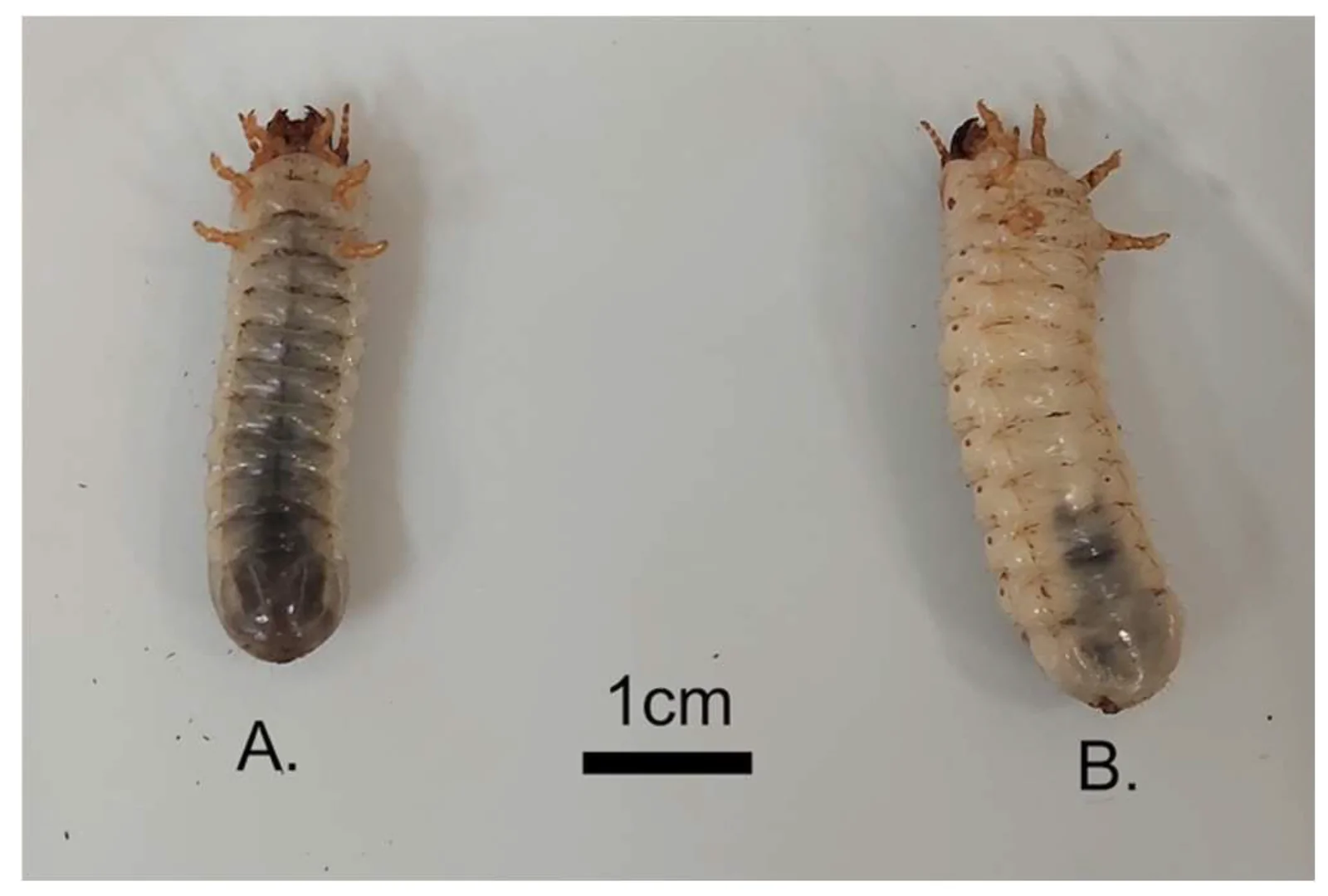
*Goliathus goliatus* larvae without (**A**) and with (**B**) koi feed supplementation of the diet to achieve adequate protein. The scale bar is 1 cm.

Table 1 shows the width, length, and body mass of the larvae. *Goliathus goliatus* were significantly longer (*t* = 5.078, *p* < 0.01), wider (*t* = 9.643, *p* < 0.001), and heavier (*t* = 6.215, *p* < 0.001) when fed the high-protein diet recommended for their development, as expected (Figure 1). *Mecynorrhinella poggei* did not significantly differ in size when fed the high-protein diet (*p* > 0.05), also as expected, though the values trended smaller in the high-protein diet. Hindgut masses did not significantly differ between the two treatments of *G. goliatus* but were significantly heavier in *M. poggei* fed the substrate without koi feed (*t* = 3.219, *p* < 0.05), suggesting they consumed more of the food.

### 3.2. Microbiome Composition and Abundance

The raw metabarcoding reads are available on the NCBI Sequence Read Archive, GenBank Accession Numbers SRR36490704-SRR36490724. A total of 1,615,633 sequence reads were obtained in the raw data (min 49,125, max 128,408, mean 76,935). Following quality control and filtering, alpha rarefaction at several sampling depths using QIIME2 confirmed that our sequencing had reached a plateau for all samples. After limiting to microbes with presence in more than one sample, QIIME2 identified 2045 unique bacterial features from the samples sent for metabarcoding, with a minimum frequency of 2899 reads per sample and a maximum of 19,622 (mean 9861). After classification and taxonomic assignment, the resulting 456 OTUs were collapsed at the maximum taxonomic level of resolution (genus) (Supplementary Table S1).

The unprocessed *Liquidambar formosana* wood’s microbiome primarily consisted of *Rummeliibacillus* sp. (Bacilli: Bacillales: Planococcaceae) (11.74% of reads), *Chryseobacterium* sp. (Bacteroidia: Flavobacteriales: Weeksellaceae) (8.88%), and *Xanthomonas* sp. (Gammaproteobacteria: Xanthomonadales: Xanthomonadaceae) (6.90%). The koi feed pellet microbiome was predominantly *Leuconostoc* sp. (Bacilli: Lactobacillales: Lactobacillaceae) (16.93%) and *Escherichia–Shigella* sp. (Gammaproteobacteria: Enterobacterales: Enterobacteriaceae) (4.69%). At the end of the experiment, the most abundant microbes in the beetle-rearing substrate made from *Liquidambar formosana* without any larvae inside were Planctomycetota OM190 (9.52%)—a large but unculturable clade about which very little is known. The most abundant microbes in substrates that had been lived in by larvae were similar regardless of treatment type, with Bacillaceae (Bacilli: Bacillales) or *Sphingobacterium* sp. (Bacteroidia: Sphingobacteriales: Sphingobacteriaceae) predominating.

The most abundant microbes in the hindguts of *Goliathus* larvae reared without koi feed—a deficient diet for this species—were an unidentifiable Tannerellaceae (Bacteroidia: Bacteroidales) (average 15.97% of reads), *Proteiniphilum* sp. (Bacteroidia: Bacteroidales: Dysgonomonadaceae) (15.55%), *Alistipes* sp. (Bacteroidia: Bacteroidales: Rikenellaceae) (11.04%), *Dysgonomonas* sp. (Bacteroidia: Bacteroidales: Dysgonomonadaceae) (7.17%), and *Desulfovibrio* sp. (Desulfovibrionia: Desulfovibrionales: Desulfovibrionaceae) (6.82%). The latter was enriched in *Goliathus* larvae fed koi feed (average 15.77% of reads) while the other abundances fell, though they were still in the top seven most common genera. Significantly more abundant in the koi-fed *Goliathus* compared to the non-koi-fed larvae were *Bacteroides* (Bacteroidia: Bacteroidales: Bacteroidaceae) (9.80% vs. 0.26%, respectively) and an unidentified member of the Rhodocyclaceae (Gammaproteobacteria: Burkholderiales) (8.13% vs. 1.40%). In the guts of *Mecynorrhinella* larvae fed low-protein diets, *Proteiniphilum* sp. was more abundant (25.74% mean abundance vs. 6.85% in protein-enriched larvae), and *Dysgonomonas* was much less abundant (1.11% vs. 47.39% in protein-enriched larvae). Notably, all of these genera were extremely rare or completely undetectable in the wood, koi feed, or initial substrate microbiomes (Supplementary Table S1).

### 3.3. Statistical Comparative Microbiome Analysis

Statistical analysis was first performed on a subset of the data containing only the hindgut samples from the four treatments (*n* = 12). Table 2 summarizes the results of pseudo-F PERMANOVA and PERMDISP on the three beta diversity metrics: Bray–Curtis, Jaccard, and weighted Unifrac. Community composition significantly differed among the four species/diet groups based on Bray–Curtis, Jaccard, and weighted UniFrac distance metrics (PERMANOVA, *p* < 0.05 for all comparisons), although pairwise PERMANOVA results found no significant differences (*p* > 0.05). PERMDISP results among the four species/diet groups were non-significant, meaning the PERMANOVA results were not driven by unequal dispersion among groups. When comparing the results by species, significant differences in hindgut microbial community between *Mecynorrhinella* and *Goliathus* were detected (PERMANOVA and PERMDISP, *p* < 0.01 for Bray–Curtis and Jaccard; *p* > 0.05 for weighted unifrac). When comparing the results by diet, no significant differences could be detected (*p* > 0.05) other than a barely significant (*p* = 0.048) Jaccard difference, meaning insects fed different diets mostly had similar microbiomes, all else (i.e., species) being equal. Pairwise comparisons of the two species with the same diet (*n* = 6) or two diets with the same species (*n* = 6) consistently showed no significant differences (*p* > 0.05).

**Figure 2:**
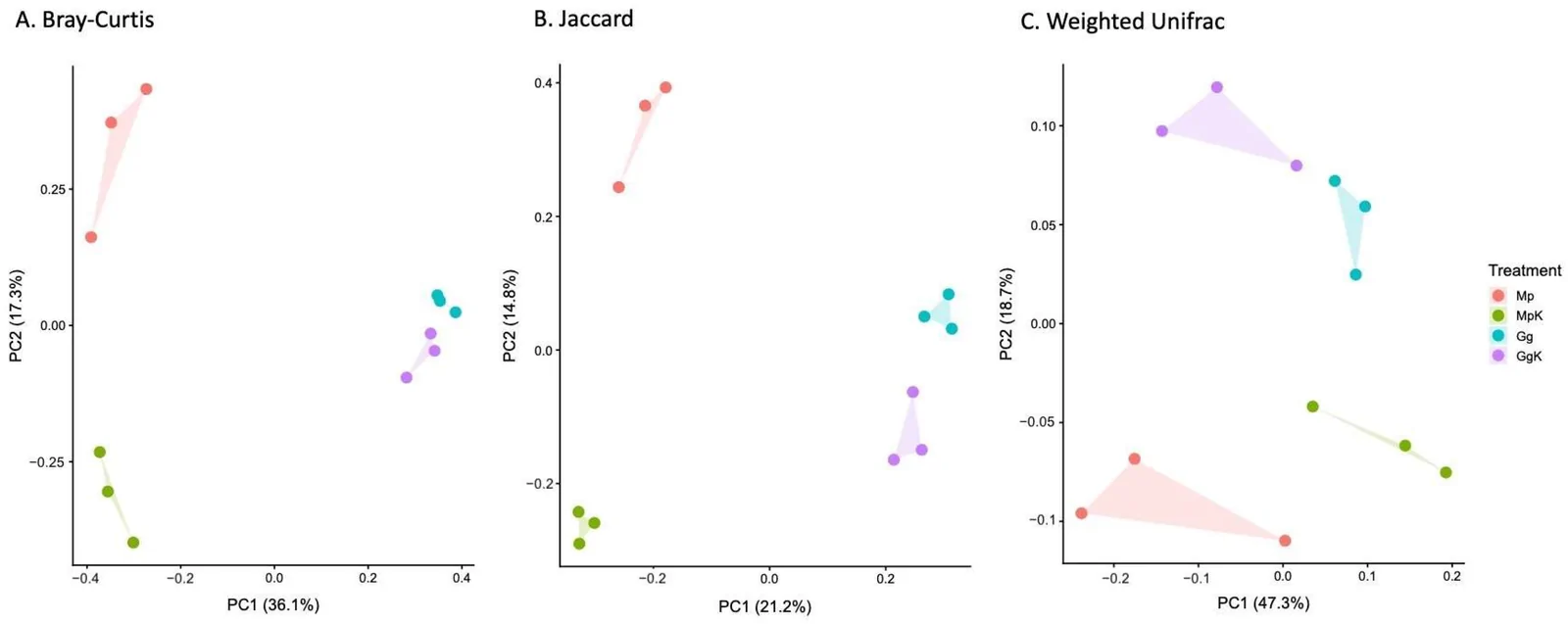
Principal Coordinate Analysis visualization of the (**A**) Bray–Curtis dissimilarity, (**B**) Jaccard beta diversity, and (**C**) weighted UniFrac metrics in microbiome diversity. Diet supplementation affected the *M. poggei* microbiomes more strongly than the *G. goliatus* microbiomes, and microbiomes overall differed more strongly by species than with diet. Treatments are coded as the species plus “K” for koi feed supplementation of the diet.

PCoA based on Bray–Curtis, Jaccard, and weighted UniFrac distances using R showed separation of microbial communities primarily by host species along the first principal coordinate, with Mp/MpK samples clustering separately from Gg/GgK samples (Figure 2). Figure 2A shows a PCoA plot of the Bray–Curtis dissimilarity, which compares communities based on relative abundance. Figure 2B shows a PCoA plot for the Jaccard beta diversity metric, which uses presence/absence rather than relative abundance and is more strongly influenced by rare taxa, and Figure 2C shows a PCoA plot for the weighted UniFrac metric that accounts for abundance and phylogeny. While the two treatments of *Goliathus* larvae cluster more closely together, each treatment group of species plus diet occupies a distinct region of multivariate space, suggesting the groups are clean. The *Goliathus* microbiomes are positive on the first principal components axis (PC1) for Bray–Curtis and Jaccard, while the two treatments of *Mecynorrhinella* larvae are negative on the PC1 axis. The same occurs along axis 2 for weighted Unifrac. In all cases, this data suggests the beetle species has a greater impact on the microbiome than the diet. The UniFrac data suggests the differences are phylogenetically structured, which matches the evidence that Dysgonomonadaceae are more abundant in *Mecynorrhinella*, while Tannerellaceae and Desulfovibrionaceae are more abundant in *Goliathus.*

ANCOM-BC analysis of the hindgut microbiomes only revealed significant differences (W > 2400, q < 0.05) for several features between the two beetle species, corresponding with the already presented data. For example, a feature identified as *Proteiniphilum saccharofermentans* was significantly upregulated in low-protein *G. goliatus* (log fold change 5.7922, q < 0.001) and significantly downregulated in *M. poggei* with low-protein (−5.8129, q < 0.001) and high-protein diets (−5.8026, q < 0.001). The complete data file is available as Supplementary Table S2

### 3.4. Predicted Microbiome Functional Analysis

**Figure 3:**
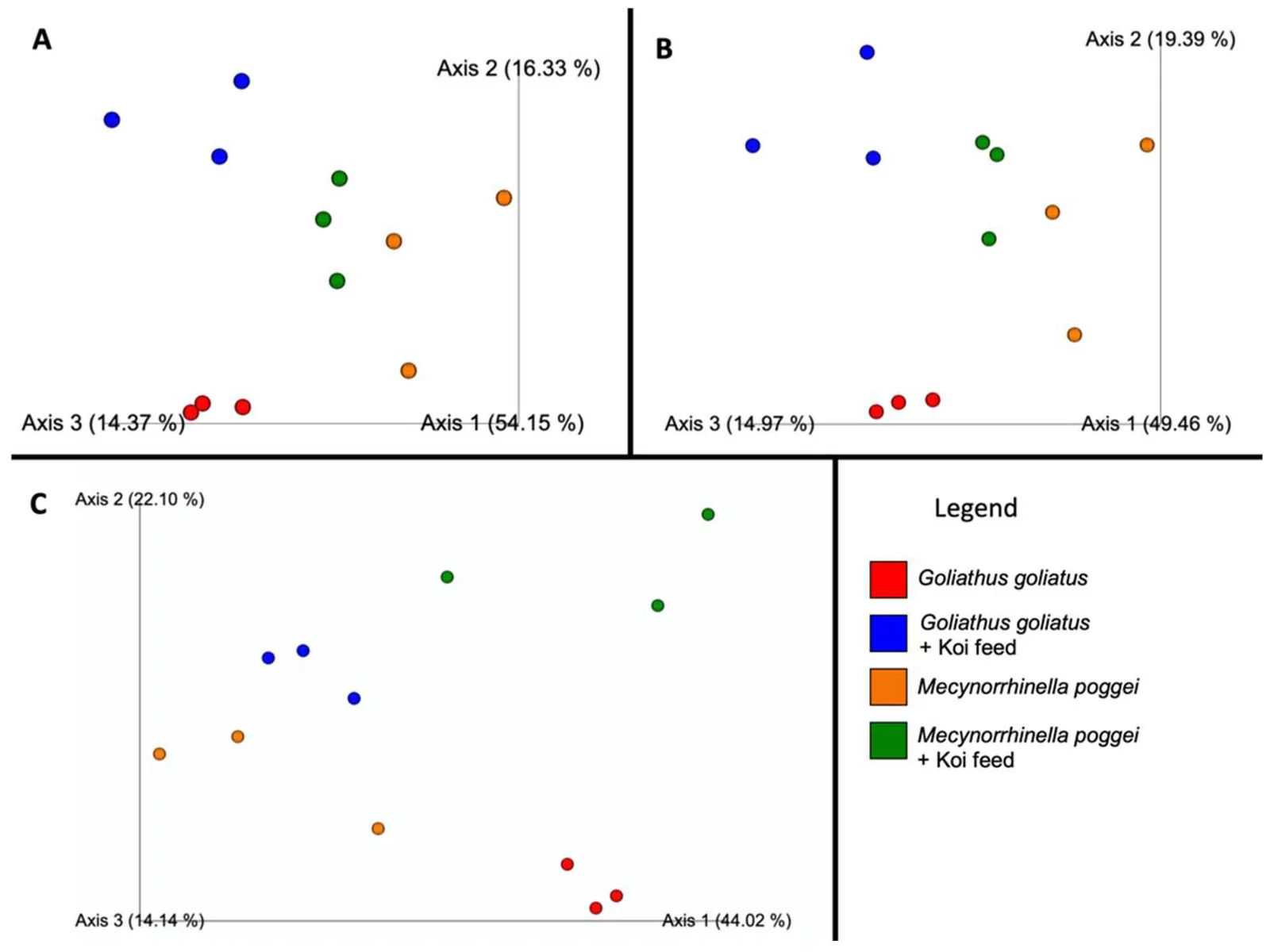
Emperor Principal Coordinate Analysis visualization of the Bray–Curtis dissimilarity for the (**A**) EC numbers, (**B**) KEGG orthologs, and (**C**) MetaCyc pathways identified by PICRUSt. The four hindgut samples were distinct but, nonetheless, clustered relatively close together.

PERMANOVA showed statistical differences in the composition and differential abundance (Bray–Curtis) of different EC numbers (Pseudo-F_12,4_ = 8.028431, *p* < 0.001), KEGG orthologs (Pseudo-F_12,4_ = 7.262328, *p* < 0.001), and MetaCyc pathways (Pseudo-F_12,4_ = 6.102989, *p* < 0.001) among the four treatment types, but no pairwise differences between beetles of the same species on different diets or beetles of different species on the same diet (*p* > 0.05 for all). PCoA plots showed that, while each treatment group’s samples clustered together, all the hindgut samples clustered together too (Figure 3). When comparing Shannon’s alpha diversity among the four treatment types only, no significant differences were found for EC numbers (Kruskal–Wallis H = 7.51282, *p* > 0.05), KEGG orthologs (H= 7.666667, *p* > 0.05), or MetaCyc pathways (H= 7.615385, *p* > 0.05).

**Figure 4:**
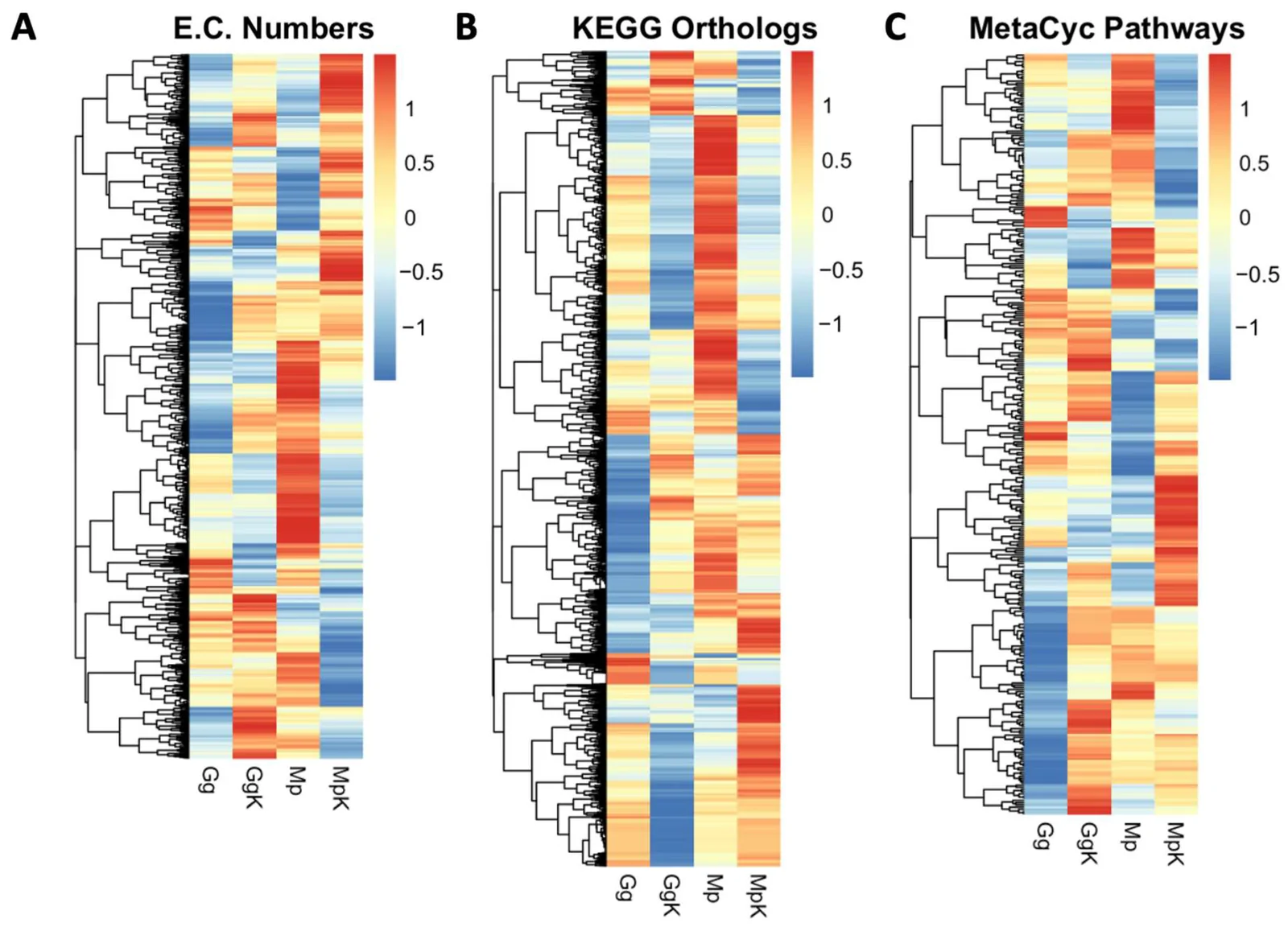
Heatmaps of the (**A**) EC numbers, (**B**) KEGG orthologs, and (**C**) MetaCyc pathways with significantly different log fold changes at q < 0.001 after ANCOM-BC analysis of the four hindgut samples. Samples labeled as in Figure 2.

Figure 4 shows the heatmaps of the EC numbers, KEGG orthologs, and MetaCyc pathways with significantly different pairwise log fold changes at q < 0.001 after ANCOM-BC of the four hindgut samples alone. The complete data files of these results are available as Supplementary Tables S3–S5, respectively. Among the hydrolases (EC 3), specifically the digestive enzymes, PICRUSt2 predicted greater expression in the *Goliathus goliatus* microbiomes of the exo-1,3-beta-glucanases (EC 3.2.1.58, q < 0.001), which degrade fungal cell walls or polysaccharides, but no other remarkable differences were attributable to species or diet. Other enzymes were more bacteria-specific, like sporulation factors (EC 3.4.21.116) and germination proteases (EC 3.4.24.78), important for bacterial sporulation but not likely beneficial to the host insect. Among digestive enzymes in KEGG, no significant differences (q > 0.05) between species or diets were found for cellulases (e.g., K01179, K01225), hemicellulases (e.g., K01181, K01226), amylases (e.g., K01182, K01187), pectinases (e.g., K01051, K01786), beta-glucanases (e.g., K01206, K01207), cell wall esterases (e.g., K01212), proteases (e.g., K01372, K01375, K01379, K01380), or peptidases (e.g., K01271, K01272, K01273), with the minor exception of a statistically significant (q < 0.01) but small (0.250 difference in log_2_ fold changes) increase in predicted alpha-amylase (K01176) expression levels in the guts of larvae with low-protein feed. Regarding nitrogen fixing, no pairwise differences between any pair of treatments were found for the nitrogen fixation marker genes nitrogenase iron protein (K02588), nitrogenase alpha subunit (K02586), and nitrogenase beta subunit (K02591). Among MetaCyc pathways, relatively few showed significant (q < 0.05) pairwise differences in predicted expression between species or between diets based on ANCOM-BC analysis of the PICRUSt2 data. The only potentially meaningful such pathway was 3-phenylpropanoate degradation (P281-PWY), as 3-phenylpropionate is naturally produced by degradation of organic matter, including plant lignin. This pathway was significantly (q < 0.001) enriched in *Mecynorrhinella poggei* (log_2_ fold change = 2.259) and depleted in *Goliathus goliatus* (log_2_ fold change = −4.84) regardless of diet.

## 4. Discussion

A limitation of this study is that the sample size was low, even if it was still within the precedents set by past published research, due to limited availability of experimental material: these are large, slow-growing, and difficult-to-acquire species. The results thus can be considered preliminary until validated by larger sample sizes, if possible. Another limitation is that only predicted functional profiles were used. While this is commonly done in microbiome studies, microbiome function verification would require metatranscriptomics or enzyme activity assays.

*Goliathus goliatus* larvae needed protein supplementation to thrive in culture, as expected, making this the first paper to officially verify the anecdotal knowledge among breeders that *Goliathus goliatus* requires extra protein. *Mecynorrhinella poggei* does not need extra protein and, in this study, showed no statistically significant improvement or worsening in development when provided extra protein. A declining trend in length and mass was observed with protein supplementation, which, if verified in a study with more individuals, could be explained in a number of hypothesized, testible ways: altered gut pH, increased ammonia production from protein fermentation, reduced feed consumption, relative lack of digestible carbohydrates, or other forms of mild stress.

This experiment’s main purpose was to test whether the microbiome of these beetles correlates more with their species and possibly therefore their inherent dietary requirements and gut physiology or with their diet, which both limits the available pool of microbes and sets the nutritional limitations the gut microbiome and insect gut must overcome. Overall the results showed that microbiomes were more similar in beetles of the same species rather than those fed the same diet. While the four species–diet combinations each had distinct microbiomes, neither species showed significantly different gut microbiomes overall when the diet was changed except for some rare taxa as suggested by the just barely significant differences in Jaccard values between both species fed protein and both species not fed protein (Table 2). This finding would suggest that the most abundant taxa on one diet remained among the most abundant when the diet changed even if their relative ranks changed. However, those differences vanished when only comparing data from one species at a time (Table 2) and may be an artifact of the interspecies differences when all four treatment types were compared across the two diets instead of each species alone.

While *Goliathus goliatus* showed measurably reduced growth on the low-protein diet, their gut microbiota relative abundance was not significantly different between treatments based on the statistical analyses (Table 2). It contained similar percentages of *Dysgonomonas*, *Proteiniphilum*, *Alistipes*, and an unidentified Tannerellaceae (all Bacteroidales). Koi feed supplementation shifted relative abundances of certain microbes like *Desulfovibrio*, but this did not lead to any significant changes in predicted microbiome functions relatable to digestion, other than slightly higher predicted exo-1,3-beta-glucanase levels. No other PCWDEs, proteinases, or nitrogen-fixing genes were predicted to be enriched in the microbiome from either diet. Based on the limited change in microbiome, *G. goliatus* can be tentatively hypothesized to have a relatively conserved, diet-resistant gut microbiome comprising the aforementioned microbes. Whether this hypothesized potential “core” is indeed found in wild specimens and across the geographic range of the beetle remains to be seen. The *Goliathus* larvae’s hindgut microbiome is thus suggested to be functionally and taxonomically resilient to protein deficiency. Whatever the potential benefits of such conservation, a cost is that the larvae were unable to recruit beneficial microbes to their gut that could potentially produce or recover more nitrogen from the deficient diet, such as nitrogen-fixing microbes. This further raises the question of whether nitrogen-fixing microorganisms are limiting in abundance or activity in the larvae, or whether microbial nitrogen fixation is simply unimportant during the larval stage. That the gut microbiome did not adapt by enriching nitrogen-fixing taxa may imply that the conserved gut community lacks the capacity for nitrogen-fixing, that the host lacks the ability to recruit such microbes, and/or that the diet in this case did not contain sufficient nitrogen-fixing microbes to recruit. Thus, the growth limitation in *Goliathus* may be attributed at least in part to the microbiome’s functional rigidity, which prevents compensatory nitrogen acquisition through biological fixation.

*Mecynorrhinella poggei*’s gut microbiome too was not significantly altered by the addition of koi feed to their diet based on the statistical analyses (Table 2), despite having measurably less *Proteiniphilum* and more *Dysgonomonas* in the high-protein diet. In this case, the protein supplementation was unnecessary and had no effect on their development. *Dysgonomonas* is a common insect gut microbe, including in wood-feeding beetle larvae [59,60], and is often capable of degrading complex polysaccharides and fermenting amino acids [61]. *Proteiniphilum* in the same family is, as its name suggests, proteinolytic and known to thrive in protein-rich environments [62]. Neither species is unexpected in insect guts [63], including scarab beetles [64,65], and there is no evidence that either reflects a dysbiosis. No PCWDEs, proteinases, or nitrogen-fixing genes were predicted to be enriched in the microbiome from either diet. Based on the limited change in taxonomic composition, *M. poggei* too can be tentatively hypothesized to have a moderately conserved gut microbiome, but, again, studies of different populations are necessary to test this hypothesis, which may prove incorrect.

The different diets, lived-in substrates, and guts were all enriched with different microbes. The gut microbiomes, where Bacteroidales dominated, could not have been predicted from knowing the microbiome of the diets, where other clades were more abundant. Rare microbes that were almost or completely undetectable in substrates like *Proteiniphilum* could be enriched, if not dominant, in certain guts, demonstrating that selection within the host is a powerful force shaping the gut microbiome. For example, the lower oxygen levels in the beetle gut would be a significant selective force, readily explaining why anaerobes like *Dysgonomonas* and *Proteiniphilum* were more abundant in the gut than in the oxygenated substrate. The substrate microbiota also changed over time compared to the unprocessed wood, and the presence of any larvae led to the enrichment of Bacillaceae. Larvae can alter the substrate microbiota through frass deposition, nutrient enrichment, and physical disturbance, overriding initial substrate community composition, as has been noted with other detritivores [60,66].

Regarding the PICRUSt predictions of potential microbiome function, few to no significant differences in digestion-related, predicted functional pathways were detected after dietary shifts in either species. This can be easily explained by the taxonomic conservation of the microbiomes: since the microbiomes did not change, their predicted functional profiles should not have changed either. It could also suggest functional conservation: the community function of the microbiomes was buffered regardless of compositional change, suggesting ecologically redundant microbes can preserve the stability of the digestive process, as was hypothesized for the related *Oryctes* beetle larvae [18,23]. Broader studies of the natural gut microbiome variability in these beetle species across their geographic and dietary ranges are needed before conclusive statements about their microbiome’s taxonomic or potential functional conservation can be made. The mechanisms by which a gut could be colonized by different yet functionally equivalent species of microbes, as in *Oryctes*, are unknown at this time: whether or not a symbiotic, non-pathogenic, free-living microbe colonizes a gut is presently not considered to be an inherent trait of the microbe or the insect gut [67]. Functional conservation could be a coincidental effect of, for example, a gut and/or diet microenvironment being most hospitable to the classes of microbes that are also most likely to have beneficial traits for the insect’s digestion. Alternatively, apparent functional conservation could be due to survivor bias, as insects whose guts were not colonized by functionally equivalent microbes upon a dietary change would not survive to have their gut microbiomes measured.

While a greater sample size would confirm this, the data suggests most of the statistically detectable differences in gut microbiome with the change in diet in these beetle larvae, if any, were due to changes in low-abundance taxa. This finding is in itself not too surprising if one posits a correlation between abundance and the ability to colonize the gut and persist within it. Many specific, mutually nonexclusive mechanisms could explain why different dietary microbes colonize different guts at different rates, such as gut oxygen and pH levels and gradients, antimicrobial peptides, gut epithelial ultrastructure, or nutrient availability within the gut lumen [67]. The presence and relative abundance of any microbe in an individual insect gut is also shaped by the microbial ecology of the substrate being consumed and the physiology of the gut at the time and further complicated by microbial antagonism and competition among the species. That said, this finding raises the question of the relative roles of abundant versus rare gut microbes toward the functional profile of the gut microbiome. Rare taxa can serve as a reservoir of functional redundancy that becomes more abundant or important when a diet changes or under stress conditions, facilitating functional conservation. Alternatively, they represent transient environmental passersby that are selectively filtered by the host and have limited functional impact compared to the community core that remains stable. The mechanisms by which the stably dominant microbes achieve both stability and dominance are, again, difficult to determine and likely mutually nonexclusive.

## 5. Conclusions

In summary, in this study, host species was the primary determinant of hindgut microbiome structure. The most abundant gut microbes in both *Goliathus* and *Mecynorrhinella* remained so in response to dietary manipulation, and the predicted functional profiles of the microbiomes remained conserved in all treatments. The adaptive benefits of this functional conservation are not fully clear, as *Goliathus* is protein-dependent while *Mecynorrhinella* is not. They may reflect limitations on what the scarab gut is physiochemically capable of being colonized by from the environment. The myriad hypotheses on what determines the composition of a gut microbiome and how it impacts host phenotype require more tests of diverse populations of different organisms to be evaluated.

## Figures and Tables

**Table 1:** Sizes (mean ± SD, *n* = 4) of scarab larvae with and without a protein-supplemented diet. Superscripted letters indicate the means for *Goliathus goliatus* differed between the two treatments. No such difference between treatments was found for *Mecynorrhinella poggei*.

Insect	Diet	Length (mm)	Width (mm)	Mass (g)
*Goliathus goliatus*	w/o koi feed	315 ± 25 ^b^	7.80 ± 0.38 ^b^	1.35 ± 0.20 ^b^
	w/koi feed	405.25 ± 25 ^a^	10.27 ± 0.34 ^a^	2.68 ± 0.37 ^a^
*Mecynorrhinella poggei*	w/o koi feed	425 ± 12	10.32 ± 0.12	3.34 ± 0.15
	w/koi feed	390 ± 40	10.51 ± 1.07	2.85 ± 0.54

**Table 2:** Results of pseudo-F PERMANOVA and PERMDISP comparing the hindgut microbiomes across species–diet treatment combinations, between the two species, between the two diets, and between the species and diet pairs on the three beta diversity metrics, Bray–Curtis, Jaccard, and weighted Unifrac. Gg = *Goliathus goliatus*. Mp = *Mecynorrhinella poggei*. K = supplemented with Koi feed for extra protein. *n* = sample size (three per diet/species pair).

Mp|MpK|Gg|GgK (*n* = 12)	PERMANOVA			PERMDISP	
Metric	Pseudo-F	*p*	Pairwise	F	*p*
Bray–Curtis	4.215344	0.001	all > 0.05	0.995298	>0.05
Jaccard	2.174088	0.001	all > 0.05	0.448721	>0.05
Weighted Unifrac	4.962171	0.002	all > 0.05	1.015381	>0.05
Mp + MpK vs. Gg + GgK (*n* = 12)	PERMANOVA			PERMDISP	
Metric	Pseudo-F		*p*	F	*p*
Bray–Curtis	5.562789		0.005	8.896548	0.004
Jaccard	2.657150		0.004	7.858418	0.003
Weighted Unifrac	2.160661		>0.05	1.664627	>0.05
Gg + Mp vs. GgK +MpK (*n* = 12)	PERMANOVA			PERMDISP	
Bray–Curtis	1.625291		>0.05	0.023785	>0.05
Jaccard	1.572630		0.048	0.786577	>0.05
Weighted Unifrac	1.783286		>0.05	0.01424	>0.05
Gg vs. GgK (*n* = 6)	PERMANOVA			PERMDISP	
Bray–Curtis	2.877780		>0.05	0.697181	>0.05
Jaccard	1.518662		>0.05	0.023313	>0.05
Weighted Unifrac	5.678071		>0.05	2.596757	>0.05
Mp vs. MpK (*n* = 6)	PERMANOVA			PERMDISP	
Bray–Curtis	2.509249		>0.05	0.154903	>0.05
Jaccard	1.928833		>0.05	0.540059	>0.05
Weighted Unifrac	5.269307		>0.05	0.199227	>0.05
Gg vs. Mp (*n* = 6)	PERMANOVA			PERMDISP	
Bray–Curtis	4.961509		>0.05	2.283902	>0.05
Jaccard	2.155821		>0.05	0.878405	>0.05
Weighted Unifrac	6.126533		>0.05	1.898979	>0.05
GgK vs. MpK (*n* = 6)	PERMANOVA			PERMDISP	
Bray–Curtis	4.794715		>0.05	0.393485	>0.05
Jaccard	2.405037		>0.05	0.077664	>0.05
Weighted Unifrac	5.306014		>0.05	0.007205	>0.05

## Data Availability

The original data presented in this study are openly available in the NCBI Sequence Read Archive, GenBank Accession Numbers SRR36490704-SRR36490724.

## Supplementary Materials

The following supporting information can be downloaded at: https://www.mdpi.com/article/10.3390/insects17080871/s1, Table S1: The microbes identified in the *Goliathus* and *Mecynorrhinella* digestive tracts plus their substrates as per QIIME 2 binning and identification against the Silva 138 99% OTUs full-length sequences [47,48], collapsed to the genus level; Table S2: ANCOM-BC analysis [45,55] presenting the log fold change and its significance (q value) in the abundance of each feature in the four treatment groups (*Goliathus* and *Mecynorrhinella* with and without koi feed added to the diet) for different microbe OTUs; Table S3: ANCOM-BC analysis [45,55] showing log_2_ fold changes and corresponding q-values for predicted abundances of each EC number across four treatment groups (*Goliathus* and *Mecynorrhinella*, each with and without koi feed supplementation), as inferred using PICRUSt2 [56]; Table S4: ANCOM-BC analysis [45,55] showing log_2_ fold changes and corresponding q-values for predicted abundances of each KEGG orthology across four treatment groups (*Goliathus* and *Mecynorrhinella*, each with and without koi feed supplementation), as inferred using PICRUSt2 [56]; Table S5: ANCOM-BC analysis [45,55] showing log_2_ fold changes and corresponding q-values for predicted abundances of each MetaCyc pathway across four treatment groups (*Goliathus* and *Mecynorrhinella*, each with and without koi feed supplementation), as inferred using PICRUSt2 [56].

## Abbreviations

The following abbreviations are used in this manuscript:

ANCOM-BC	Analysis of Compositions of Microbiomes with Bias Correction
EC	Enzyme Commission
KEGG	Kyoto Encyclopedia of Genes and Genomes
OTUs	Operational taxonomic units
PCoA	Principal Coordinate Analysis
PCWDE	Plant cell wall-degrading enzyme
