# Supplementary material for: Effect of Protein Supplementation on the Gut Microbiome of Omnivorous and Herbivorous Goliath Beetles
Source: Insects. 2026 Aug 21;17(8):871. doi: 10.3390/insects17080871 (PMC13512936; doi:10.3390/insects17080871)
Supplement: Supplementary file 1 [file insects-17-00871-s001.zip › Supplementary Data Table Captions.pdf]

Supplementary Data Table S1: The microbes identified in the *Goliathus* and *Mecynorrhinella* digestive tracts plus their substrates as per QIIME 2 binning and identification against the Silva 138 99% OTUs full-length sequences [47,48], collapsed to the genus level. Values are the number of reads of that feature per sample. Koi: initial koi feed pellets (triplicate, pooled). Wood: Initial *Liquidambar formosana* substrate (triplicate, pooled). S: Substrate sample from rearing containers without larvae (duplicate, pooled). SGL: Substrate used to rear *Goliathus* larvae (duplicate, pooled). SGLK: Substrate with koi feed supplementation used to rear *Goliathus* larvae (duplicate, pooled). SML: Substrate used to rear *Mecynorrhinella* larvae (duplicate, pooled). SMLK: Substrate with koi feed supplementation used to rear *Mecynorrhinella* larvae (duplicate, pooled). Gg#: Hindguts of individual *Goliathus* larvae from substrate diet. GgK#: Hindguts of individual *Goliathus* larvae from koi feed supplemented substrate diet. Mp#: Hindguts of individual *Mecynorrhinella* larvae from substrate diet. MpK#: Hindguts of individual *Mecynorrhinella* larvae from koi feed supplemented substrate diet.

Supplementary Data Table S2: ANCOM-BC analysis [45,55] presenting the log fold change and its significance (q value) in the abundance of each feature in the four treatment groups (*Goliathus* and *Mecynorrhinella* with and without koi feed added to the diet) for different microbe OTUs. Species were identified as against the Silva 138 99% OTUs full-length sequences [47,48], and the top hit is provided. Samples are labeled as follows. Koi: The initial koi feed pellets (triplicate, pooled). Wood: The initial *Liquidambar formosana* substrate (triplicate, pooled). S: Substrate sample from rearing containers without larvae (duplicate, pooled). SGL: Substrate used to rear *Goliathus* larvae (duplicate, pooled). SGLK: Substrate used to rear *Goliathus* larvae with koi feed supplementation (duplicate, pooled). SML: Substrate used to rear *Mecynorrhinella* larvae (duplicate, pooled). SMLK: Substrate used to rear *Mecynorrhinella* larvae with koi feed supplementation (duplicate, pooled). Gg#: Hindguts of individual *Goliathus* larvae from substrate diet. GgK#: Hindguts of individual *Goliathus* larvae from koi feed supplemented substrate diet. Mp#: Hindguts of individual *Mecynorrhinella* larvae from substrate diet. MpK#: Hindguts of individual *Mecynorrhinella* larvae from koi feed supplemented substrate diet

Supplementary Data Table S3: ANCOM-BC analysis [45,55] showing $\log_2$ fold changes and corresponding q-values for predicted abundances of each EC number across four treatment groups (*Goliathus* and *Mecynorrhinella*, each with and without koi feed supplementation), as inferred using PICRUSt2 [56]. Samples are labeled as in Supplementary Data Table S2.

Supplementary Data Table S4: ANCOM-BC analysis [45,55] showing $\log_2$ fold changes and corresponding q-values for predicted abundances of each KEGG orthology across four treatment groups (*Goliathus* and *Mecynorrhinella*, each with and without koi feed supplementation), as inferred using PICRUSt2 [56]. Samples are labeled as in Supplementary Data Table S2.

Supplementary Data Table S5: ANCOM-BC analysis [45,55] showing $\log_2$ fold changes and corresponding q-values for predicted abundances of each MetaCyc pathway across four treatment groups (*Goliathus* and *Mecynorrhinella*, each with and without koi feed supplementation), as inferred using PICRUSt2 [56]. Samples are labeled as in Supplementary Data Table S2.
